# Vehicle Trajectory Prediction with Lane Stream Attention-Based LSTMs and Road Geometry Linearization

**DOI:** 10.3390/s21238152

**Published:** 2021-12-06

**Authors:** Dongyeon Yu, Honggyu Lee, Taehoon Kim, Sung-Ho Hwang

**Affiliations:** Department of Mechanical Engineering, Sungkyunkwan University, 2066 Seobu-ro, Suwon 16419, Korea; yunotions2@skku.edu (D.Y.); ghdrb9138@skku.edu (H.L.); th94228@skku.edu (T.K.)

**Keywords:** trajectory prediction, LSTM encoder–decoder model, attention mechanism, road geometry, autonomous driving

## Abstract

It is essential for autonomous vehicles at level 3 or higher to have the ability to predict the trajectories of surrounding vehicles to safely and effectively plan and drive along trajectories in complex traffic situations. However, predicting the future behavior of vehicles is a challenging issue because traffic vehicles each have different drivers with different driving tendencies and intentions and they interact with each other. This paper presents a Long Short-Term Memory (LSTM) encoder–decoder model that utilizes an attention mechanism that focuses on certain information to predict vehicles’ trajectories. The proposed model was trained using the Highway Drone (HighD) dataset, which is a high-precision, large-scale traffic dataset. We also compared this model to previous studies. Our model effectively predicted future trajectories by using an attention mechanism to manage the importance of the driving flow of the target and adjacent vehicles and the target vehicle’s dynamics in each driving situation. Furthermore, this study presents a method of linearizing the road geometry such that the trajectory prediction model can be used in a variety of road environments. We verified that the road geometry linearization mechanism can improve the trajectory prediction model’s performance on various road environments in a virtual test-driving simulator constructed based on actual road data.

## 1. Introduction

Intelligent vehicles, including partially automated vehicles that are equipped with Adaptive Cruise Control (ACC), require the ability to drive strategically according to the flow of traffic while simultaneously ensuring safety. Strategic driving includes determining when to change lanes between surrounding vehicles, passing low-speed or erratically behaving vehicles, and creating space for surrounding vehicles to change lanes. Autonomous vehicles must have the ability to predict the future behaviors and trajectories of surrounding vehicles to implement these features. Predicting surrounding vehicles’ behaviors is a core element that has a significant effect on everything from planning trajectories for basic driving [1,2] to high-level features such as predictive control for improving comfort and safety [3] and high fuel efficiency driving [4]. The ability to predict the future behavior of surrounding vehicles requires sensing technology that accurately recognizes obstacles [5] and interacts with surrounding autonomous driving systems [6].

To date, several methods for predicting vehicle trajectories have been proposed. The classic trajectory prediction method that is generally employed uses a Bayesian filtering technique such as a Kalman filter in the vehicle motion model [7,8,9]. These methods use simple models to ensure quick computation speed and are good at predicting the near future; however, they show poor performance regarding long-term predictions that reflect the nonlinear movements of vehicles. To address these limitations, more elaborate models such as the Gaussian mixture model [10] and Dynamic Bayesian Network (DBN) [11] have been proposed. Nevertheless, they have not been sufficient for depicting the various nonlinear dynamic motions of actual vehicles. Laugier et al. proposed a method for predicting a future path using the Hidden Markov Model (HMM), which probabilistically models the change in a specific state [10,11]. Schreier et al. presented a method to improve long-term prediction performance by designing a Bayesian network to classify vehicle behavior and predict detailed routes [12].

Recent studies have used various deep learning methods for trajectory prediction. These studies have mainly used the Recurrent Neural Network (RNN) technique to learn and predict time series data [12]. The challenge of predicting vehicle trajectories, in which a vehicle’s future position is predicted based on a series of past data, has aspects in common with the work on voice recognition and Natural Language Processing (NLP), which have garnered success in the field of machine learning. The Long Short-Term Memory (LSTM) model, which resolves the basic RNN model’s vanishing gradient challenge, has exhibited excellent performance in the field of time series data learning [13,14,15,16]. Studies have been presented that have improved long-term prediction by applying the benefits of the LSTM model to the problem of sequence-to-sequence prediction [17] of vehicle trajectories [18,19,20,21] or by applying them to dynamic obstacles such as pedestrians [22].

**Figure 1:**
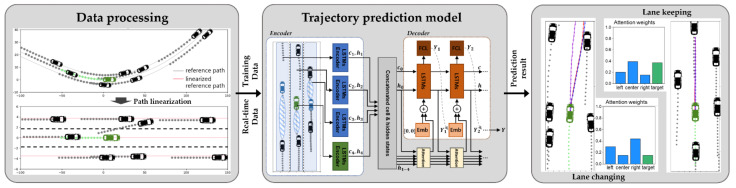
Framework of the proposed vehicle trajectory prediction method.

This study proposes methods to improve the vehicle future trajectory prediction model’s learning efficiency and long-term prediction performance. The driving conditions of surrounding vehicles were transformed from the perspective of the predicted target vehicle, and an attention mechanism was applied to the LSTM model to selectively focus on important information. In order to apply the model developed from the traffic dataset to the natural environment, we propose a method of transforming a complex road shape into a simplified straight frame. The framework of the proposed vehicle trajectory prediction method is illustrated in Figure 1, and it comprises the two parts below.

Section 3 presents a lane stream attention-based LSTM encoder–decoder model. This inputs information related to surrounding vehicles and outputs the future coordinates of the target vehicles. It is based on a local coordinate system that is fixed on the rear wheel surface center of the target vehicle. This method does not simply input all the position information related to surrounding vehicles in a specified area around the target vehicle; instead, it summarizes the information as data that depict the adjacent lane’s traffic stream information and the target vehicle’s main status. It is possible to improve learning efficiency and long-term prediction performance by configuring an attention mechanism that can focus on each situation among the context vectors of each lane and target vehicle state. This study used the Highway Drone (HighD) dataset [23], which is a large-scale, naturalistic traffic vehicle trajectory dataset, to train the model.

Section 4 presents a road geometry linearization method for effectively using the trajectory prediction model on roads with various geometries. Most previous studies have developed trajectory prediction models based on data obtained from straight roads and then evaluated the performance on these road sections. Therefore, it is unlikely that the reported performance will be achieved when using these models in autonomous vehicles that must drive on real roads with various geometries. A road geometry linearization method was developed by noting that actual drivers determine their driving intentions based on the longitudinal or lateral positions of surrounding vehicles within each lane rather than on the relative positions of surrounding vehicles. This study verifies that it is possible to obtain sufficient trajectory prediction performance, even on curved roads, when the proposed linearization method is used.

## 2. Related Work

The various proposed vehicle trajectory prediction methods can be classified according to the interactions between the surrounding vehicles and the target vehicle [20,24]. Recent related studies using deep learning models to effectively improve long-term prediction performance are summarized as follows.

**Independent prediction:** Initial studies on vehicle trajectory prediction calculated the independent movement of the target vehicle based on vehicle kinematic or dynamic modeling. Kalman filters are mainly used to track the vehicles’ positions and predict their future states [7,8,9,25,26]. Trajectory prediction based on simple physical models and Kalman filters has the disadvantage of only being effective at predicting future states for a short time. Gaussian mixture modeling [10] or Monte Carlo path planning [27] is used to solve the short-term prediction problem. Efforts have been made to improve prediction performance by classifying the future behavior of vehicles before making physical state predictions. Finite behavior types are classified using behavior classification models, such as Bayesian networks [28], support vector machines [29,30], and HMM [31,32,33], and trajectories or risks corresponding to each behavior are predicted.

**Interaction aware prediction:** Interaction aware prediction methods consider interactions between vehicles. A relatively small number of such works have been published. Optimal predictions regarding future motions have been made using heuristic cost functions [34,35,36], data-driven random forest classifiers [37], and Markov decision processes [38] based on relative information between vehicles. In [34], 10 types of maneuvers are classified using HMM, and an Interacting Multiple Model (IMM) is used to model the vehicles’ motion. An energy minimization cost function is applied to the results of a maneuver recognition module and a trajectory prediction module for each vehicle, and this is used as a module for predicting interactions. The use of optimization models has a limitation in that these models are significantly affected by how well the cost function is designed. In another method, the results of classifying lateral maneuvers using a random decision forest model based on data obtained in an actual road environment are combined with a Gaussian mixture regression model’s probabilistic prediction trajectories [37]. Approaches based on real road data have the burden of needing to construct a large-scale dataset, and they may be overfitted for certain situations if data from a variety of driving environments cannot be obtained.

**Recurrent networks-based prediction:** Mozaffari et al. published a detailed investigation into deep learning-based vehicle behavior prediction [39]. Convolutional Neural Networks (CNNs) are used to predict surrounding vehicles’ driving intentions and trajectories based on sensor data [40,41,42,43,44]. As CNN-based prediction methods lack a mechanism for reflecting time-series information, researchers have presented works that use RNNs and CNNs in combination to integrate the advantages of each model [19,45,46,47]. Researchers have also proposed a method that uses occupancy grid maps and an LSTM encoder–decoder model to probabilistically predict trajectories [18]. In [19,20], interactions with surrounding vehicles are modeled by a social pooling mechanism. The context vectors of trajectory and maneuver encoders are combined to predict the maneuver-specific future distributions of vehicles’ positions [48]. The LSTM RNN model can be used for intelligent traffic management and route guidance by predicting traffic flow from a macro perspective [49] as well as microscopic vehicle movement trajectory prediction. A traffic control system that considers current and future traffic congestion conditions has improved a city’s traffic flow [50]. Classification is performed on behaviors such as lane changes and deceleration, which can be recognized by turn signals and brake lights in real road situations. In Neural Machine Translation (NMT), which is an important field that uses the sequence-to-sequence model, researchers have published results in which translation performance and learning efficiency are greatly improved by using an attention mechanism that selectively focuses on parts of the source text [51,52]. In the field of vehicle trajectory prediction, researchers have also begun to use attention mechanisms to improve prediction performance by focusing on information such as certain vehicles or time points [21,53,54,55]; however, additional research is still required on various methodologies that can effectively emulate the methods of judgment used by actual drivers. In most related studies, the future vehicle trajectory prediction models are trained and verified based on the Next Generation Simulation (NGSIM) [56,57] or HighD [23] datasets. Nevertheless, these studies have not sufficiently verified their performance on a variety of road geometries. Yoon et al. predicted realistic driving intentions by extracting road geometry data from detailed roadmap data and then using the extracted data as constraints in a prediction model [58].

## 3. Proposed Vehicle Trajectory Prediction Method

The vehicle trajectory prediction model was designed based on dynamics data such as the relative positions and speeds of the surrounding vehicles converted to the reference frame of the target vehicle. First, a basic LSTM encoder–decoder model was designed, and an attention mechanism was applied to increase the ability to understand the driving context of the encoder.

### 3.1. Problem Formulation

Our task was to predict the future trajectories of surrounding vehicles in various driving environments; this has an important effect on the performance of partially or fully automated vehicles. This study utilized the idea of a deep learning model, which has caused performance improvements compared to past efforts in fields such as NLP and NMT; these have the common point of predicting continuous future data based on a series of past data. For the performance of a prediction algorithm, providing high-quality, large-scale data is as important as the structure of a deep learning model. Fortunately, we could utilize public datasets containing the driving information of actual traffic vehicles [23,55,56]. The deep learning model’s learning efficiency and prediction accuracy are improved by organizing the data in the dataset, which contains vast amounts of information, to prioritize the information that mainly affects the decisions of actual drivers. Another challenge is to acquire versatility such that the prediction ability, which is limited to the learning dataset’s driving environment, can be used in a variety of situations. We addressed this challenge by noting that human drivers would consider vehicles driving along a predetermined road geometry similarly to vehicles driving straight.

### 3.2. Surrounding Vehicle Data Processing

**Figure 2:**
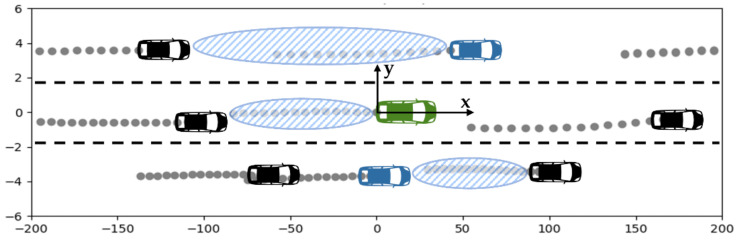
Coordinate system used for trajectory prediction. Green: target vehicle, blue: nearest vehicle in adjacent lane, patterned ellipse: front and rear inter-vehicle distance.

We converted the data for surrounding vehicles based on a local coordinate system that was fixed on the center of the back face of the target vehicle, as illustrated in Figure 2 (Figure 2 shows some of the driving data used for learning). When human drivers or autonomous vehicles perceive a driving situation and interact with surrounding vehicles, they think from the perspective of a local coordinate system with their own field of view as the reference point. If global coordinates expressed the positions of vehicles, the data described by the numbers would be completely different to the target vehicle’s viewpoint, even in the same situation. This could cause inefficiencies in learning and harm prediction performance.

In Figure 2, the green vehicle’s heading direction is the *x*-axis and the direction perpendicular to that is the *y*-axis. The blue vehicles are the closest cars in each lane adjacent to the target vehicle, and they are the objects that must be examined with the greatest caution when considering a lane change. The traffic flow of each lane is expressed by the distance between the front and rear vehicles and their amount of change, as well as the location and speed of the ego (green) vehicle and the blue vehicles.

Tijerina et al. observed that lane changing durations last an average of 5.0 s on city streets and 5.8 s on highways [59]. Toledo et al. analyzed the duration of lane changes according to variables such as vehicle type, driving velocity, and relative distance. [60]. Previous studies, including [61,62], have reported lane change durations of approximately 5 s on average; therefore, we predicted the position of the target vehicles up to 5 s into the future based on information on the target vehicles and surrounding vehicles from the past 3 s.

### 3.3. Base LSTM Encoder–Decoder Trajectory Prediction Model

**Figure 3:**
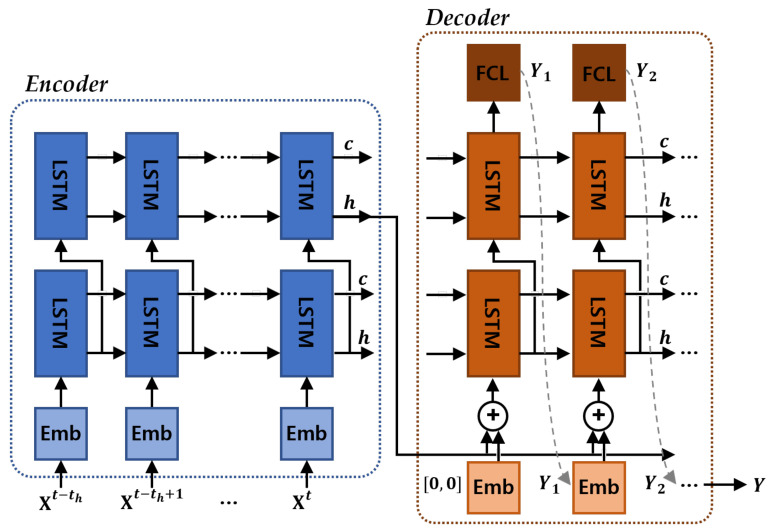
Peaky LSTM encoder–decoder architecture.

LSTM encoder–decoder models have been proposed in the field of machine translation [14,63]. To verify our proposed model’s performance and the effectiveness of the learning data configuration, the peaky LSTM encoder–decoder model illustrated in Figure 3 was created by referencing architecture [14] that was proposed in the field of machine translation. Decent prediction performance can be expected, even from a basic model, if the model uses large amounts of data that properly depict the situations to be learned [64,65].

- Input and output data: Our model’s input comprised the data related to the traffic flow of the target vehicle’s lane as well as the lanes to the left and right of the target vehicle for a fixed amount of time.

(1) $$X=\left[x^{t-t_{h}},x^{t-t_{h}+1},\;\ldots ,\;x^{t-1},\;x^{t}\right]$$

Here,

(2) $$x^{t}=\left[x_{c}^{t},\;x_{l}^{t},\;x_{r}^{t}\right],$$

(3) $$x_{c}^{t}=\left[x_{m}^{t},\;y_{m}^{t},v_{x,m}^{t},\;v_{y,m}^{t},\;x_{f}^{t},\;y_{f}^{t},d_{mf}^{t},\;\Delta v_{mf}^{t},x_{r}^{t},\;y_{r}^{t},\;d_{mr}^{t},\;\Delta v_{mr}^{t}\right]\;$$

Data from the current time t until the time before $t_{h}$ were adopted as the input and data were sampled from up to 3 s before, at 5 Hz. The value $x^{t}$ at time t is the center, left, and right lane data (c: center, l: left, r: right), and each lane’s data included the center vehicle’s *x*- and *y*-axis position, velocity, distance to the vehicles in front and behind, and relative velocity (m: middle, f: front, r: rear). The center vehicles of each lane were the green or blue vehicles illustrated in Figure 2.

The model’s output was the future coordinate of the target vehicle after time $t_{f}$.

(4) $$Y=\left[y^{t+1},y^{t+2},\;\ldots ,\;y^{t+t_{f}}\right]$$

Here,

(5) $$y^{t}=\left[x_{tgt}^{t},\;y_{tgt}^{t}\right]$$

**Figure 4:**
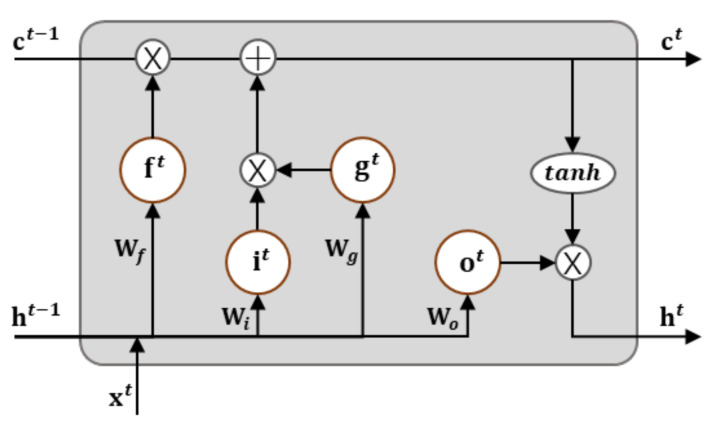
The structure of an LSTM cell.

- Encoder: The encoding layer receives data from each time as the input and sends it through the embedding and LSTM layers to convert it to hidden state vectors. The cell and hidden state vectors that are calculated in the LSTM for each time step are sent to the next step. The topmost LSTM layer’s hidden state at the final time point acts as the context vector in which the driving information of the vehicles for a fixed amount of time is encoded. The LSTM has memory cells that summarize the past input sequences and store them, and these cells consist of the following gating mechanisms that properly combine the new input and memory information (Figure 4).

(6) $$forget,f=\;\sigma \left(x^{t}W_{xf}+h^{t-1}W_{hf}+b_{f}\right)$$

(7) $$input,i=\;\sigma \left(x^{t}W_{xi}+h^{t-1}W_{hi}+b_{i}\right)$$

(8) $$update,g=\;\tanh\left(x^{t}W_{xg}+h^{t-1}W_{hg}+b_{g}\right)$$

(9) $$output,\;o=\;\sigma \left(x^{t}W_{xo}+h^{t-1}W_{ho}+b_{o}\right)$$

(10) $$cell\;state,c^{t}=f⊙c^{t-1}+g⊙i$$

(11) $$hidden\;state,h^{t}=o⊙\tanh(c^{t})$$

where $\sigma \left(x\right)$ is an activation function, $W_{x}$ and $W_{h}$ represent weight matrix for input and hidden state, $b_{f}$ is a bias vector of forget gate, and $c^{t}$ and $h^{t}$ denote cell and hidden state vectors at time step t.

- Decoder: The context vectors that summarize the past driving information are sent from the encoding layer to the decoder layer’s input. The hidden states that are calculated in the LSTM layer for each time step are converted to x and y coordinates by the fully connected neural network layer. The position vectors that are ultimately produced as an output become the input of the next step, and a similar process is repeated until the goal prediction time is reached to determine a continuous future prediction position at each time point. The peaky LSTM encoder–decoder model connects the position vectors that become the input of each time step and the context vectors that are produced as an output by the encoding layer. The prediction performance can be improved by not sending the context vector solely on the decoder’s first step and then using the past driving information at each step.
- Loss function: In deep learning models, training progresses in the direction of reducing the loss function. Our ultimate training goal was to determine prediction points at the closest distance to the actual future position. Therefore, we adopted the Root Mean Square Error (RMSE), which corresponds to distance error, as the loss function. Additionally, more importance was placed on lateral accuracy than longitudinal accuracy [21].

(12) $$RMSE=\sqrt{\frac{1}{n}\overset{n}{\underset{i=1}{\sum }}\overset{t_{f}}{\underset{t=1}{\sum }}\begin{array}{c} \left\{{\left(\hat{x}-x\right)}^{2}+2\cdot {\left(\hat{y}-y\right)}^{2}\right\} \end{array}}$$

Here, x, y are the true values at each time step, and $\hat{x},\hat{y}$ are the predicted positions.

### 3.4. Lane Stream Attention-Based LSTM Encoder–Decoder Trajectory Prediction Model

In the NMT field, the input information that is summarized as context vectors of fixed length is considered to cause an obstruction in improving the performance of the encoder–decoder architecture. Performance is improved by introducing a mechanism that can focus on the most relevant information in the context vector at each prediction time point [51,52]. In the challenge of vehicle trajectory prediction, unlike that of translation, it is effective to focus on information such as traffic lanes’ driving flow rather than certain time points in the input information [21].

**Figure 5:**
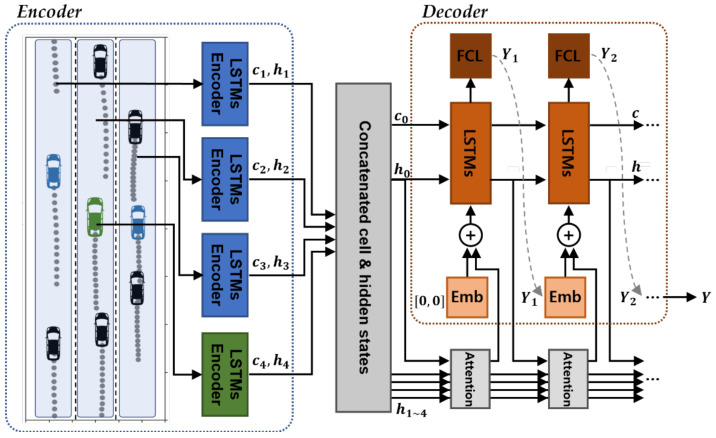
Lane stream attention-based LSTM encoder–decoder architecture.

Our proposed lane stream attention-based LSTM encoder–decoder model was created by adding an attention mechanism to the basic model that separates and encodes the driving flow of each lane, as well as the target vehicle information, and determines its degree of importance at each time step, as described in Section 3.3 (Figure 5).

- Input and output data: The proposed attention-based model uses three encoders to depict the driving flow of the lanes that are adjacent to the target vehicle and one encoder that focuses on the target vehicle. The input data comprise central information that is considered with great caution when a human driver adjusts the vehicle’s velocity or changes lanes [66,67]. The lane driving flow encoder’s input is

(13) $$x_{lane}^{t}=\left[x_{m}^{t},\;y_{m}^{t},v_{x,m}^{t},\;v_{y,m}^{t},\;x_{f}^{t},\;y_{f}^{t},d_{mf}^{t},\;\Delta v_{mf}^{t},x_{r}^{t},\;y_{r}^{t},\;d_{mr}^{t},\;\Delta v_{mr}^{t}\right]\;$$

and the target vehicle encoder’s input is

(14) $$x_{tgt}^{t}=\left[x_{tgt}^{t},\;y_{tgt}^{t},v_{x,tgt}^{t},\;v_{y,tgt}^{t},\;x_{front}^{t},\;x_{rear}^{t},\;x_{left}^{t},\;x_{right}^{t},\;s_{turn},s_{brake}\right].$$

The lane information input at time t is similar to those of the peaky LSTM model’s lane input data. The target vehicle information input includes the *x*- and *y*-axis positions, velocity, and longitudinal positions of the surrounding vehicles in the front, back, left, and right directions. In addition, the states of the turn signals and brake lights are added as they provide the most important hints when an actual human driver predicts a surrounding vehicle’s movement. As brake lights generally come on unconditionally when the brake pedal is pressed, they are set to the “on” state when decelerating by more than a certain velocity change. An average of 52–75% of actual drivers use turn signals in situations such as turning at intersections or changing lanes [68,69]. We set the turn signals to the “on” state during 60% of lane-change sequences in the training data. The model’s output is the target vehicle’s future position coordinates after time $t_{f}$.

- Attention: The final hidden state that is produced as an output by each encoder summarizes the sequence of driving information, and the influence of the most recent information is strongly reflected; therefore, it is appropriate to use this as a context vector that predicts the future position. The weight of each context vector is calculated as follows:

(15) $$hidden\;states,\;h_{0}=\;\mathrm{concat}\left(h_{1},\;h_{2},\;h_{3},\;h_{4}\right)$$

(16) $$a_{n}^{t}=\;\mathrm{FCNN}\left(\mathrm{concat}\left(h^{t},h_{n}\right)\right),\;n=1,\;2,\;3,\;4$$

(17) $$attention\;weights,\;A^{t}=\;\mathrm{softmax}\left(\mathrm{concat}\left(a_{1}^{t},a_{2}^{t},a_{3}^{t},a_{4}^{t}\right)\right)$$

The context vector $h^{t}$ at prediction time t is a hidden state vector that includes the past information and the predicted position in the LSTM layer, and the initially inputted $h_{0}$ is connected to the final hidden states that are produced as outputs by each encoder. A neural network was utilized to quantify the importance of the encoding layer’s output $h_{1–4}$, and a softmax function was used to normalize the values to between 0 and 1. The hidden state vectors were multiplied by the weights and added to the embedded input to become the next step’s LSTM input value. The method for calculating the attention weights was developed by referencing models proposed by previous studies on machine translation and trajectory prediction [21,52].

- Encoder–decoder: The encoding and decoding layers are similar to those described in the basic LSTM structure. However, in an attention-based model, a total of four encoding layers are utilized and a context vector that reflects the attention weights is added to the decoding layer. To prevent the challenge of the model becoming overfitted to the training data in the training process, the data are scaled and dropout is applied to the LSTM layer.

## 4. Evaluation of Vehicle Trajectory Prediction Model with Traffic Dataset

This section presents the training results of the proposed trajectory prediction model using a natural traffic dataset. The data required for learning were extracted from publicly available datasets, and additionally necessary values were subjected to a pre-processing process. For proper performance evaluation, prediction results of different models using the same dataset were compared.

### 4.1. HighD Traffic Dataset

**Figure 6:**
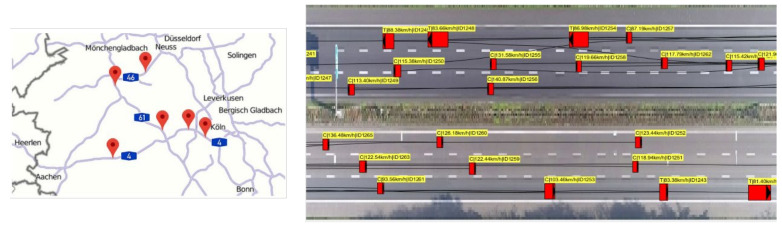
HighD dataset: highway traffic dataset with drone [23].

We used the HighD dataset, which includes publicly available traffic data, to train and evaluate the proposed model [23]. The HighD dataset is a large-scale, naturalistic vehicle trajectory dataset that includes driving data from more than 110,000 cars and trucks captured by drones on highways in Germany (Figure 6). A total of 16.4 h of data were captured from six different road sections, and the length of each section was approximately 420 m. The videos were recorded at 25 fps in 4K resolution, and a computer vision algorithm was used to automatically extract the vehicles’ data. The extracted information on each vehicle included the size, type, driving direction, position, velocity, acceleration, number of lane changes, and IDs of surrounding vehicles. The advantage of the HighD dataset is that positions are measured with an error of less than 10 cm from a vast amount of high-quality raw data. Additionally, various types of information, such as the surrounding vehicles’ information and lane changes, have been preprocessed. As it already includes various information that describes driving situations, it is very efficient for configuring data for learning.

We extracted the input data for each vehicle that was required for training and added information such as turn signals. The target vehicle and surrounding vehicles’ position and velocity data were all converted to the standard local coordinate system illustrated in Figure 2. If there was an empty position among eight vehicles around the target, the relative position and speed could not be calculated, so it was replaced with virtual data from 300 m that did not affect driving. To reduce unnecessary burden on the prediction model, 25 fps data were down-sampled to 5 fps, and the data that were divided into evenly spaced intervals were used to obtain lane-changing sequences. We divided the preprocessed dataset into training (70%), validation (10%), and testing (20%) sets, and then performed the training.

### 4.2. Training Results of Trajectory Prediction Model

The proposed model predicts future positions at up to 5 s into the future at 5 Hz. We compared the actual future positions and prediction results at 1 s intervals using the RMSE metric to verify the model’s prediction performance. Table 1 compares the results of several baseline models that were published using a similar dataset. The baseline models were divided into groups comprising models that use only the position data of the vehicles and models that also use additional information such as velocity and maneuvers. The models that learn using solely position data include Convolutional Social (CS)-LSTM (a social encoder–decoder using convolutional pooling [19]), Non Local Social (NLS)-LSTM (which combines local and non-local operations for social pooling [20]), and Multi Head Attention (MHA)-LSTM (which uses multi-head dot product attention [54]). The models that also use additional information such as vehicle velocity, acceleration, and class include MHA-LSTM (MHA-LSTM with additional features [54]), Encoder Decoder (ED)-LSTM (a basic LSTM encoder–decoder model), P-LSTM (peaky LSTM encoder–decoder model), ED-LSTM with CS (which uses the convolutional pooling concept [19]), and Lane Stream (LS)-LSTM (the proposed model in this study, which uses lane stream attention).

As presented in Table 1 and Table 2, the models that learn solely from position information show relatively low performance, and it is effective to use additional information such as velocity to accurately predict future positions. This is because moment-to-moment relative positions between vehicles, as well as each vehicle’s dynamics, have an important effect. If the surrounding vehicles’ data are converted to a local frame that is fixed on the target vehicle, as was performed in our proposed method, excellent prediction results can be expected from a P-LSTM model that inputs the context vectors that have been converted by a basic encoder–decoder model at each time step. The proposed LS-LSTM model’s long-term prediction performance was excellent compared to the other baseline models. This approach uses a mechanism of primarily processing and inputting the driving situation in terms of the surrounding lanes’ driving flow and the target vehicle’s information and then learning the importance of each item of information; it can be observed that this mechanism was effective.

**Figure 7:**
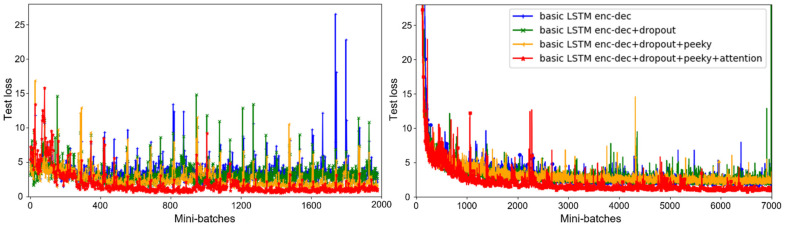
Learning curves: test and training loss.

We compared the models’ learning curves to verify the learning effectiveness of the proposed attention mechanism (Figure 7). When the attention mechanism is applied, the learning speed increases and, ultimately, a low test loss is reached in a stable manner that is clearly distinct from the other models. The peaky mechanism inputs the encoder’s context vectors in the decoder at each moment and, when it is not used, the loss becomes sufficiently low for the training dataset. However, the accuracy was poor for the test data.

**Figure 8:**
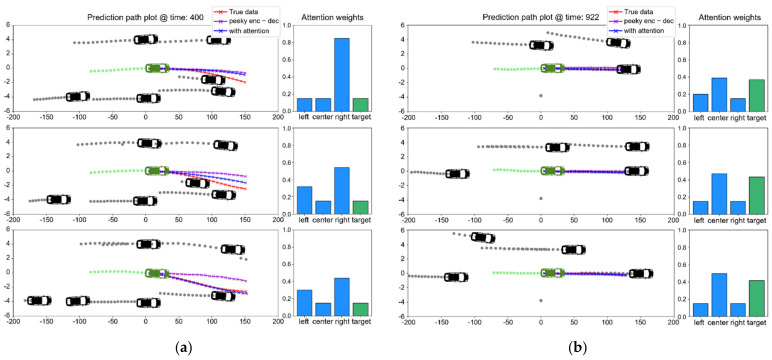
Examples of trajectory prediction: (**a**) lane changing and (**b**) lane keeping.

Figure 8 illustrates the lane change and lane-keeping sequences when using the trained model on the test dataset. In the lane change sequence in Figure 8a, the LS-LSTM model predicted the lane change more quickly than the P-LSMT model and reached the actual future trajectory. The attention mechanism calculates the weights for objects that must receive focus in real-time according to the surrounding vehicles’ information and the target vehicle’s data. In the lane-keeping sequence in Figure 8b, it can be observed that the center lane and the target vehicle’s weight values were relatively high.

## 5. Road Geometry Linearization Method for Trajectory Prediction in Real Driving Environments

**Figure 9:**
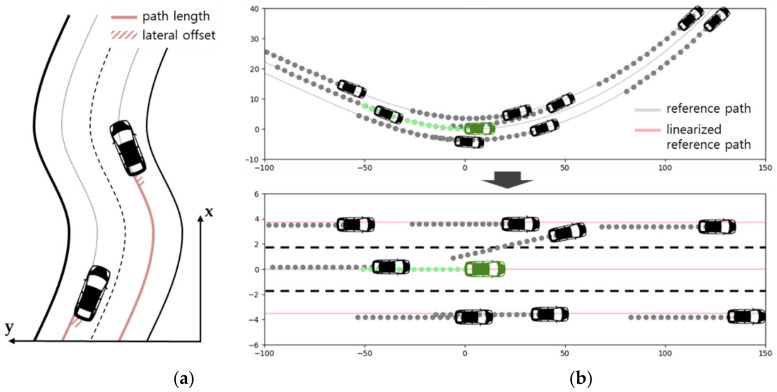
Road geometry linearization method: (**a**) reference of frame and (**b**) path linearization.

In order to expand the scope of the dataset limited to straight roads, a simulation environment was built based on actual road map data. A traffic event generation model that changes each vehicle’s longitudinal and lateral behavior over time was applied to collect traffic data in various situations in a virtual urban environment. This section proposes a method to simplify complex driving situations by linearizing a curved road’s reference path, as shown in Figure 9.

### 5.1. Road Geometry Linearization Method

The future trajectory prediction model, which was developed using a highway traffic driving dataset, exhibited excellent performance. However, most actual driving is undertaken on roads with a variety of geometries rather than completely straight roads. Therefore, to apply the trajectory prediction model to actual driving situations, one of two methods is necessary. The first is to collect driving data from roads with sufficiently different geometries and have the deep learning model consider geometry when learning. The second is to simplify the driving environment so that it is similar to a straight road scenario [70]. We used a method that linearized the road geometry, as illustrated in Figure 9, so that the proposed trajectory prediction model could function in a variety of environments.

When human drivers drive on a road such as the one illustrated in Figure 9a, the driver of the vehicle on the left side does not think that the vehicle on the right side is changing lanes to the left but rather thinks that it is moving straight along a regularly shaped road. This means that when a surrounding vehicle’s intentions are judged, how the vehicle is moving longitudinally and laterally in reference to each lane’s center is more important than the vehicle’s absolute position or heading direction. Therefore, we linearized the driving situations as illustrated in Figure 9b by using the surrounding vehicles’ progress distance and lateral offset regarding a reference trajectory corresponding to the center line of each lane. The surrounding vehicles were rotationally transformed to the local standard coordinate system fixed on the center of the back face of the target vehicle, as illustrated in Figure 2.

(18) $$\left[\begin{array}{c} x\\ y \end{array}\right]=\left[\begin{array}{cc} \cos\left(\psi \right) & -\sin\left(\psi \right)\\ \sin\left(\psi \right) & \cos\left(\psi \right) \end{array}\right]\left[\begin{array}{c} x\\ y \end{array}\right]$$

Here, ψ is the target vehicle’s yaw angle. The linearized position’s x value is the longitudinal progress distance based on the point at which the rotationally transformed surrounding vehicle’s driving lane reference trajectory crosses the *y*-axis. The linearized position’s y value is calculated by adding the spacing between lanes and the distance of lateral divergence from the reference trajectory.

### 5.2. Complex Traffic Driving Data Generation in Simulation Environment

**Figure 10:**
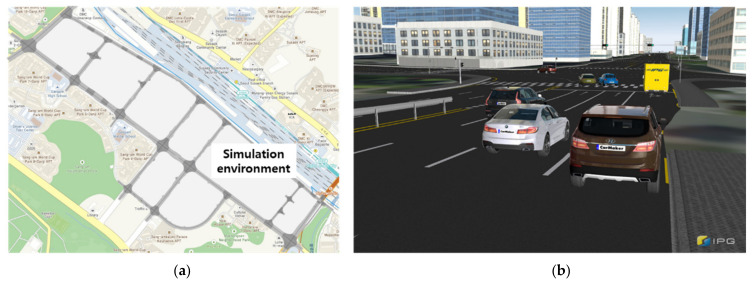
Virtual test-driving environment based on real road data: (**a**) simulation environment and (**b**) virtual driving scene.

The IPG CarMaker 10.1 virtual test-driving environment was used to acquire complex driving data, including curves and left/right turning sections, to verify the trajectory prediction model. This commercial software provides features that can realistically implement static environments, such as road models, buildings, and traffic signals, as well as dynamic driving scenarios such as traffic vehicles and pedestrians. IPG CarMaker is used for vehicle design and verification by car manufacturers or for autonomous driving algorithm development by research institutes. We configured the traffic vehicles in a simulation environment based on actual road data provided by IPG Automotive Korea and acquired the driving data. The Sangam autonomous vehicle test-driving district in Seoul, South Korea, was modeled in a virtual environment, as illustrated in Figure 10a. Figure 10b illustrates a scene from the simulation used to acquire the data.

**Figure 11:**
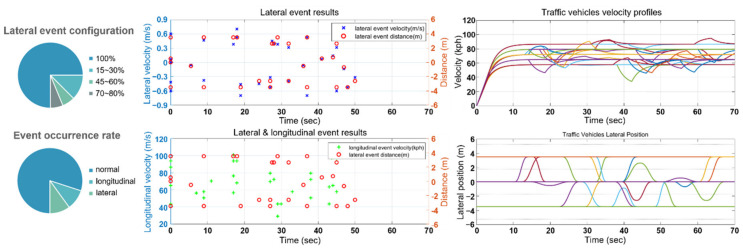
Traffic event generation results.

To create various driving scenarios with the traffic vehicles, we designed a traffic maneuver generation model (Figure 11). Each of the traffic vehicles were assigned normal, longitudinal, and lateral events at fixed ranges of time intervals. During normal events, the vehicles drive along the selected trajectory at a fixed velocity. When longitudinal or lateral events occur, the vehicles change velocity or move laterally. The traffic maneuver generation model controls the probability of each event occurring as well as the amount of acceleration/deceleration and the lateral movement distance and velocity.

The variables determined by lateral events are the lateral offset and duration. When the lateral offset is smaller than the lane width, the vehicles do not change lanes completely but move to the left or right and then return to the existing lane to model actual vehicles moving in reference to the lane center.

## 6. Experimental Evaluation

This section presents the evaluation of the proposed road geometry linearization method and trajectory prediction model based on the traffic scenarios of the virtual driving simulation. The same trajectory prediction model was applied to compare whether the route straightening model could convert curved driving data similarly to the HighD dataset driving environment.

### 6.1. Evaluation of Trajectory Prediction Model with Path Linearization Method

By using the traffic maneuver generation model, the ratio at which the vehicles change lanes can be controlled. Datasets that corresponded to the following three scenarios in the Sangam Digital Media City (DMC) virtual driving environment were created:

- Scenario 1: Driving on a straight road section that is approximately 1.2 km long with lane changes (a driving environment like that in the HighD dataset).
- Scenario 2: Repeatedly driving on a complex, closed-loop road section that is approximately 1.5 km long and has straight road and curved road sections and intersections (left and right turns) without lane changes.
- Scenario 3: Driving on a similar road to the one described in Scenario 2 with lane changes.

Table 3 presents the results of applying the model that was trained using the HighD dataset after preprocessing the acquired data using the method in Section 3.

The results of Scenario 1, a scenario similar to a highway driving environment, are similar to the training results in Table 2. The longitudinal prediction performance was reduced because the acceleration and deceleration regions caused by the longitudinal events assigned to the simulation vehicles were more rapid than the actual highway driving data. However, the lateral prediction performance was better because of the continuous and accurate position and velocity data in the simulation. The results of Scenario 1 indicate that it is important to use a variety of velocity and acceleration data in the training. Additionally, it is necessary to support an algorithm that precisely recognizes and tracks vehicles.

**Figure 12:**
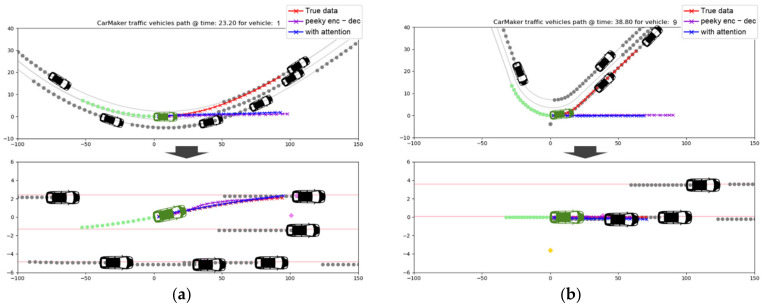
Trajectory prediction with simulation data: (**a**) lane changing and (**b**) lane keeping.

In Scenarios 2 and 3, in which vehicles drive on complex roads that include curves and intersections, when the linearization method is not used, a significant error occurs to the extent that the future trajectory cannot be meaningfully predicted. By using the trajectory linearization method, the lateral prediction results were improved so that they were similar to those of the straight road sections, as illustrated in Figure 12. On the complex roads’ curves and intersections, a fairly large error occurred in the longitudinal direction despite trajectory linearization because the vehicles’ velocities changed with relatively large acceleration.

### 6.2. Discussion

By using the traffic maneuver generation model, the ratio at which the vehicles change lanes could be controlled. Datasets that corresponded to the following three scenarios in the Sangam DMC virtual driving environment were created. This work performed long-term trajectory prediction of surrounding vehicles in a general road environment to improve the capabilities of autonomous vehicles. The framework of the proposed trajectory prediction method was configured as shown in Figure 1. The results of training and testing the trajectory prediction model using the HighD dataset are presented in Table 1 and Table 2. The long-term trajectory prediction performance was stably improved by applying the attention mechanism to the relative information of the target vehicle and surrounding vehicles in adjacent lanes. A road geometry linearization method was introduced to develop existing studies that were limited to the dataset environment of straight highways. Table 3 presents the trajectory prediction results based on the path linearization mechanism in the complex driving simulation traffic data. The prediction error was reduced by 76.7 percent by applying the proposed path linearization method in the complex driving scenario (Scenario 3). The deep learning model trained with the publicly available dataset could be applied to various road environments by simplifying the complex driving environments with the proposed trajectory prediction algorithm.

As road geometry linearization was applied based on the original reference path, it is essential to correctly determine the reference driving path of surrounding vehicles. The reference path of vehicles driving along curved roads was simplified to each lane’s middle line in this work. In order to accurately convert a complex natural driving environment into a straightened frame, a follow-up study is needed to find a reference route that vehicles generally travel on according to the curvature of the road and the driving speed or traffic direction. For example, most drivers may drive out-in-out for driving efficiency on certain roads, intentionally biasing one side to adjust the spacing with adjacent lanes, or the route itself may be complex, such as a merging section.

## 7. Conclusions

This study presented an LS attention-based LSTM encoder–decoder model and a road shape linearization method for predicting the future trajectory of surrounding vehicles. When changing lanes or adjusting speed, drivers consider relative information with surrounding vehicles; each of them is given importance. The proposed attention mechanism could implement the driver pattern by selectively focusing on adjacent lanes and the target vehicle to predict the future trajectories of vehicles. We verified the proposed model in terms of learning speed and prediction accuracy when using test data corresponding to 20% of the entire dataset. The attention mechanism determined the focused object in real-time according to the driving situation and improved the long-term prediction performance. In addition, the road geometry linearization method was applied so that the learning model that was developed based on straight road data could be used in various driving environments. The linearization mechanism was implemented in the same way real drivers perceive they are going straight when driving along a curved road shape. Traffic driving scenarios were implemented in virtual test-drive environments based on real road data, and the trajectory prediction algorithm was verified. The performance of the proposed trajectory prediction model was verified by comparing it with models using the same dataset. The improved predictive performance of the path linearization method on curved roads was evaluated in a simulated complex traffic scenario. The importance of our proposed trajectory prediction method is summarized as follows:

- The proposed lane stream attention-based trajectory prediction model improved long-term prediction accuracy by 25.4% compared to other methods. The ability to transform the context of each driving situation of the attention mechanism applied to the encoder–decoder model can predict the long-term trajectory more accurately.
- The proposed road shape linearization method simplifies the complex real road situation and expands the application range of the trajectory prediction model. In the complex traffic scenario acquired in the virtual driving environment, the distance error of the trajectory prediction model with the path linearization method was reduced by 76.7% compared to the result without the method. It is a more efficient and realistic method that can be applied to autonomous vehicles that drive on real roads rather than building large-scale traffic datasets on roads of numerous shapes.

The proposed method evaluated the accuracy in a virtual driving environment, including a curved road and a HighD dataset. Although the road shape linearization method can simplify the curved road, the prediction distance error for vehicles traveling with velocities and accelerations outside the range of the model training data was increased. In particular, the prediction accuracy for a situation in which the speed was suddenly reduced or stopped while entering an intersection that was not in the training data was lowered. In the future, to apply the predictive model to urban driving or slow-moving situations, we plan to use a combination of datasets such as NGSIM in the lower speed range for training purposes.

## Figures and Tables

**Table 1:** RMSEs in meters over a 5 s prediction horizon for the proposed and baseline models.

Prediction Horizon (s)	Position-Based Methods			Position + Other Features-Based Methods				
	CS-LSTM	NLS-LSTM	MHA-LSTM	MHA-LSTM(+f)	ED-LSTM	P-LSTM	ED-LSTM +CS	LS-LSTM
1	0.22	0.20	0.19	0.06	0.30	0.30	0.32	0.30
2	0.61	0.57	0.55	0.09	0.50	0.43	0.61	0.38
3	1.24	1.14	1.10	0.24	0.76	0.60	0.98	0.45
4	2.10	1.90	1.84	0.54	1.08	0.88	1.45	0.60
5	3.27	2.91	2.78	1.18	1.48	1.26	1.99	0.88

**Table 2:** Longitudinal and lateral RMSEs in meters.

Prediction Horizon (s)	Longitudinal Position				Lateral Position			
	ED-LSTM	P-LSTM	ED-LSTM +CS	LS-LSTM	ED-LSTM	P-LSTM	ED-LSTM +CS	LS-LSTM
1	0.25	0.28	0.31	0.28	0.18	0.08	0.09	0.09
2	0.39	0.40	0.58	0.36	0.31	0.15	0.17	0.13
3	0.62	0.56	0.95	0.41	0.44	0.22	0.26	0.18
4	0.92	0.83	1.41	0.54	0.56	0.30	0.36	0.25
5	1.32	1.19	1.94	0.81	0.67	0.39	0.46	0.33

**Table 3:** Longitudinal and lateral RMSEs in meters with simulation scenarios.

Prediction Horizon (s)	Scenario 1		Scenario 2		Scenario 3	
	P-LSTM (Long, Lat)	LS-LSTM (Long, Lat)	LS-LSTM w/o Linearization	LS-LSTM w/ Linearization	LS-LSTM w/o Linearization	LS-LSTM w/ Linearization
1	0.35, 0.09	0.37, 0.07	0.84, 0.39	0.80, 0.06	0.67, 0.48	0.73, 0.07
2	0.60, 0.15	0.68, 0.12	1.21, 1.47	1.02, 0.10	1.10, 1.75	1.14, 0.13
3	0.80, 0.20	0.75, 0.16	1.65, 3.12	1.37, 0.11	1.45, 3.69	1.54, 0.16
4	1.12, 0.24	0.83, 0.18	2.28, 5.28	1.54, 0.13	2.17, 6.26	1.83, 0.19
5	1.50, 0.28	0.91, 0.20	3.56, 7.87	1.96, 0.13	3.35, 9.37	2.31, 0.22
